# Supplementary material for: Highly Enhanced Enzymatic Activity of Mn-Induced Carbon Dots and Their Application as Colorimetric Sensor Probes
Source: Nanomaterials (Basel). 2021 Nov 12;11(11):3046. doi: 10.3390/nano11113046 (PMC8625186; doi:10.3390/nano11113046)
Supplement: Supplementary file 1 [file nanomaterials-11-03046-s001.zip › nanomaterials-1423225-supplementary.pdf]

nanomaterials

Supplementary Material

Highly Enhanced Enzymatic Activity of Mn-Induced Carbon Dots and Their Application as Colorimetric Sensor Probes

Ahyun Lee, Wooseok Kang and Jin-sil Choi *

Department of Chemical and Biological Engineering, Hanbat National University, Daejeon 34158, Korea; leeah3238@gmail.com (A.L.); gne01041@gmail.com (W.K.)
* Correspondence: jinsil.choi@hanbat.ac.kr

1. Analysis of crystal structure of the obtained Mn-assisted N-doped carbon dots (Mn:N-CDs)

The X-ray diffraction (XRD) pattern of Mn:N-CDs was obtained using a MiniFlex benchtop X-ray diffractometer (Rigaku, Japan). The broad peaks in the spectrum indicate the amorphous nature of Mn:N-CDs.

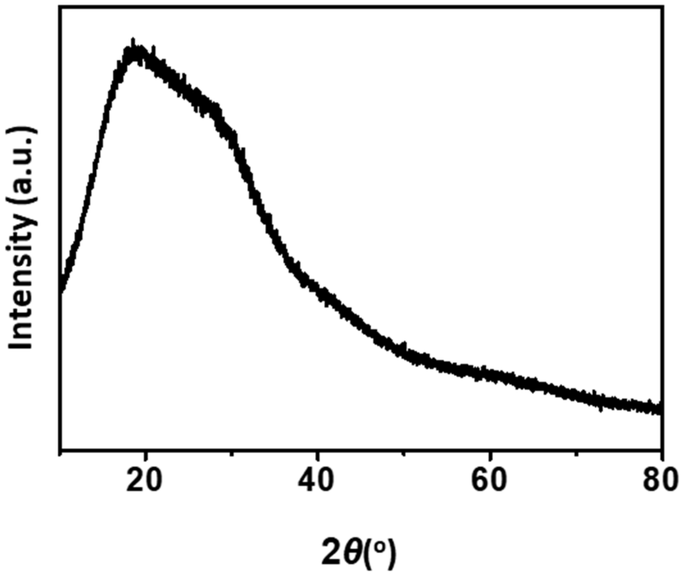

Figure S1. XRD spectrum of Mn:N-CDs.

2. Comparison of infrared (IR) spectra of N-CDs and Mn:N-CDs

The Fourier transform infrared spectra of Mn:N-CDs and N-CDs were obtained using a Nicolet 6700 (ThermoFisher Scientific, Waltham, United States). Mn:N-CDs show a higher-intensity peak at 1552 cm^{-1} (N–O stretching) than N-CDs.

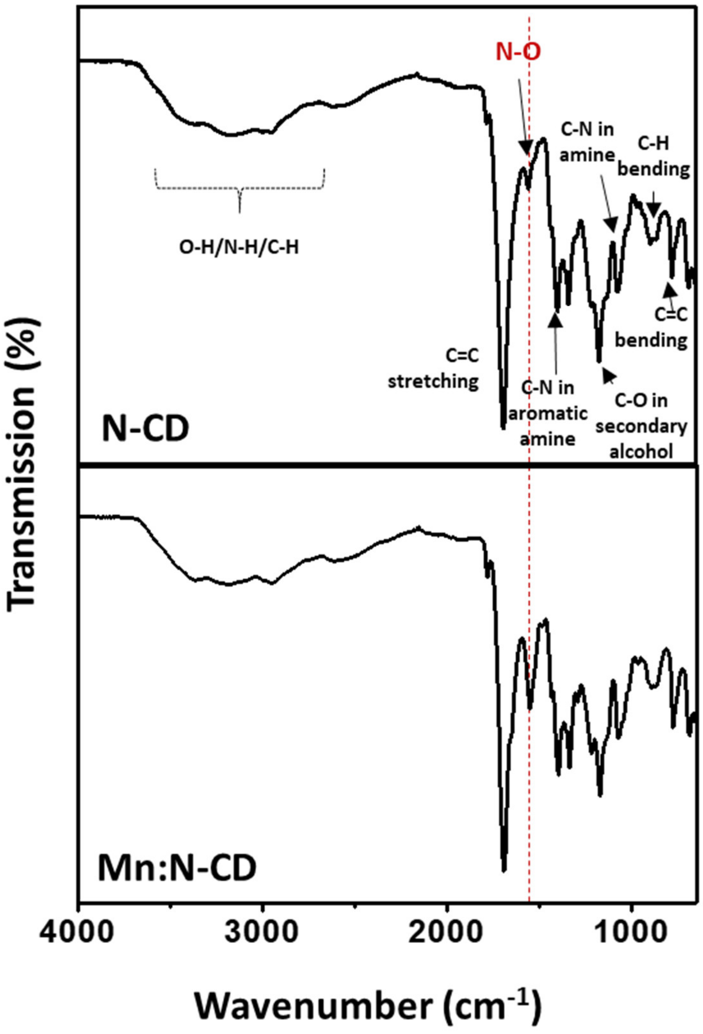

Figure S2. Comparison of IR spectra of N-CDs and as-synthesized Mn:N-CDs.

3. Analysis of the content of Mn ions in Mn:N-CDs using inductively coupled plasma-mass spectrometry (ICP-MS)

The amounts of Mn ions in three batches of Mn:N-CDs were analyzed using ICP-MS (NEXION-350X, PerkinElmer, USA), as shown in the table below.

Table S1. Amount of Mn ions in Mn:N-CDs.

Mn:N-CD	Weight (mg)	Mn^{2+} (mg)	Mn/CD (wt %)
#1	11.6	3.65290×10^{-4}	3.149×10^{-3}
#2	10.4	3.48760×10^{-4}	3.353×10^{-3}
#3	10.7	35.6533×10^{-4}	3.332×10^{-3}

4. Comparison of relative atomic compositions of N-CDs and Mn:N-CDs using X-ray photoelectron spectrometry (XPS)

The chemical compositions of N-CDs and Mn:N-CDs were investigated through XPS (K-Alpha+, ThermoFisher Scientific, USA). The relative contents of each atom and

chemical bonds were quantified by measuring the peak area in the survey scan spectra and high-resolution spectra of each atom shown in Figure 2.

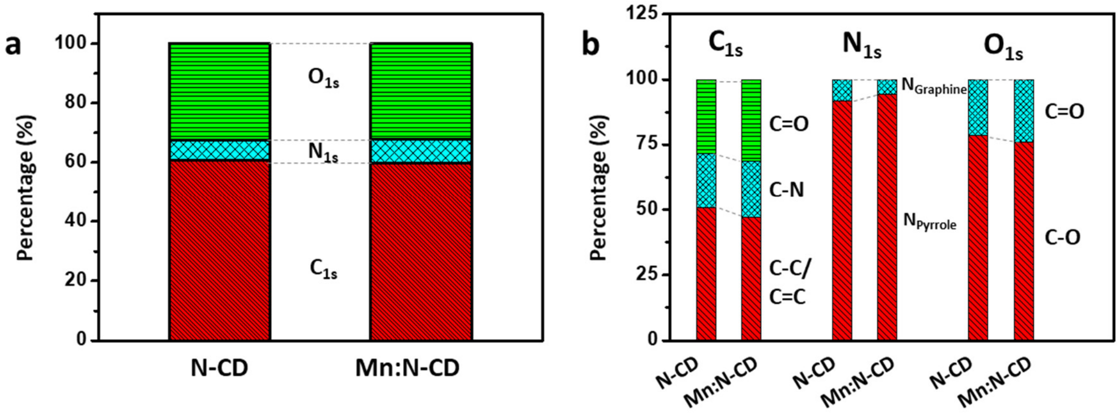

Figure S3. Ratios of (**a**) each component and (**b**) chemical bonds in N-CDs and Mn:N-CDs, which are based on XPS peak area.

5. Lineweaver–Burk plot for kinetic analysis of N-CDs and Mn:N-CDs

The linear regression curve describing the relationship between $1/V$ and $1/[S]$, viz. the Lineweaver–Burk plot, can be obtained by inverting the Michaelis-Menten equation to the following form:

$$\frac{1}{V} = \frac{K_m}{V_{max}}\frac{1}{[S]} + \frac{1}{V_{max}} \tag{1}$$

In the plot of $1/V$ vs. $1/[S]$, the ordinate and abscissa intercepts represent the inverse of V_{max} and $-1/K_m$, respectively. These were used to estimated K_m and V_{max}. Each experiment was repeated three times.

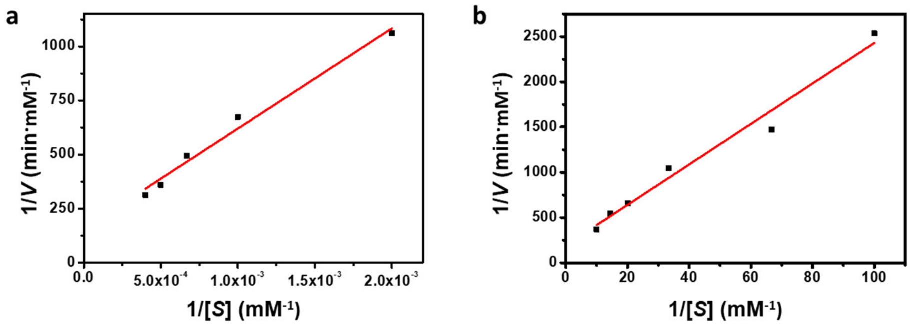

Figure S4. Lineweaver–Burk plot for the kinetic analysis of (**a**) N-CDs and (**b**) Mn:N-CDs. The concentration of 3,3',5,5'-tetramethylbenzidine was 1 mM.

6. Toxicity assay of Mn:N-CDs

The biocompatibility and cytoxicity of Mn:N-CDs were evaluated using human lung cancer cells (A549) and kidney cells (HEK293) via a 3-(4,5-dimethylthiazol-2-yl)-2,5-diphenyltetrazolium bromide (MTT) assay. Cells (2×10^5 cells per well) were cultured overnight in a 96-well microtiter plate in a 5% CO_2 atmosphere at 37 °C. The wells were filled with a cell medium containing Mn:N-CDs (25, 50, 75, 100, 250, and 500 μg mL^{-1}) and incubated for 24 h. Then, 10 μL of the MTT solution was added to each well (final concentration: 0.5 mg mL^{-1}). After the incubation of the MTT solution for 4 h, the formazan crystals, which

were formed as a result of NAD(P)H-dependent oxidoreductase in living cells, were dissolved by adding the solubilization solution from the kit. The dissolved formazan was quantified by measuring the absorbance of the solution at 550 nm using a microplate reader (SpectraMax M2e, Molecular Devices, LLC, San Jose, USA).

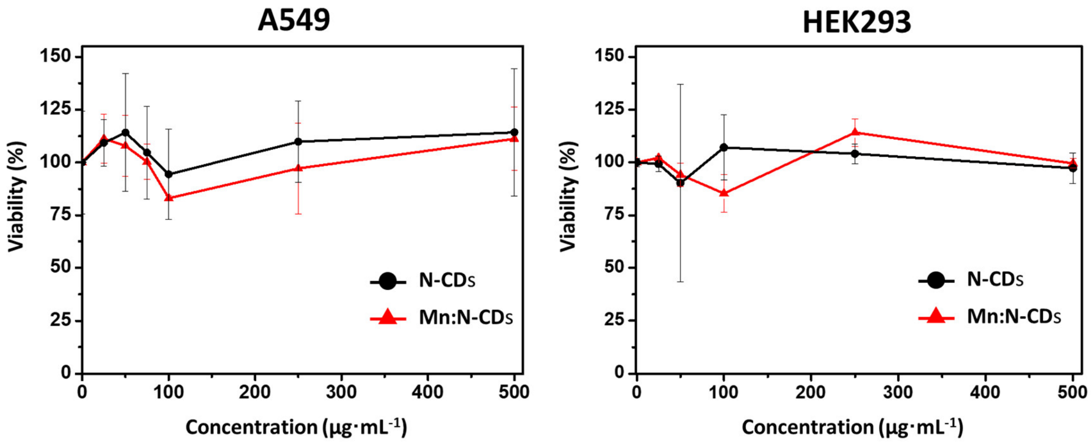

Figure S5. Viability test of N-CDs and Mn:N-CDs. Viability of A549 and HEK293 cells was examined using an MTT assay, in which cells were treated with a cell medium containing N-CDs or Mn:N-CDs for 24 h.

7. Enzymatic activity of Mn:N-CDs at various pH

The enzymatic activity of Mn:N-CDs was examined at different pH ranges from 2 to 9 Mn:N-CDs show the high enzymatic acivity at pH 2 and 3, whereas they exhibit negligible activity at pH 4 and above.

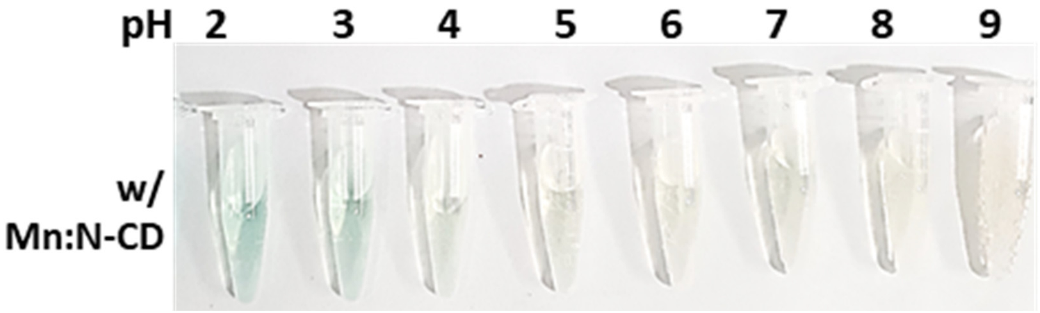

Figure S6. The image of reaction solution containing TMB, H_2O_2, and Mn:N-CDs under different pH.

8. Variation on the enzymatic activity of Mn:N-CDs when mixed with various molecules

To probe the specificity of the Mn:N-CD-based sensor system for GABA detection, the absorbance change of the reaction solution of TMB, H_2O_2, and Mn:N-CDs was monitored with the addition of citric acid, L-aspartic acid, and L-glutamic acid. When 400 nM citric acid, L-aspartic acid, and L-glutamic acid were added to the reaction solution individually, no significant change in absorbance was observed.

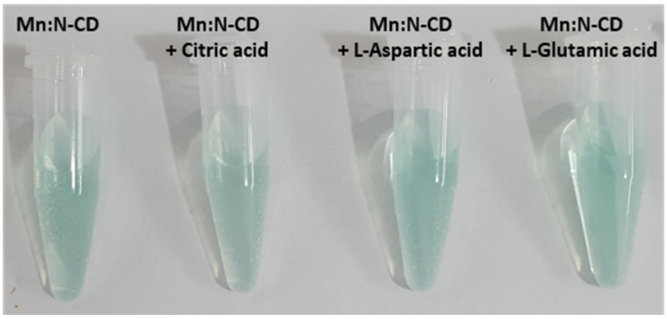

Figure S7. Image of reaction solutions that were mixed with citric acid, L-aspartic acid, and L-glutamic acid, respectively.

9. Detection of GABA in artificial urine

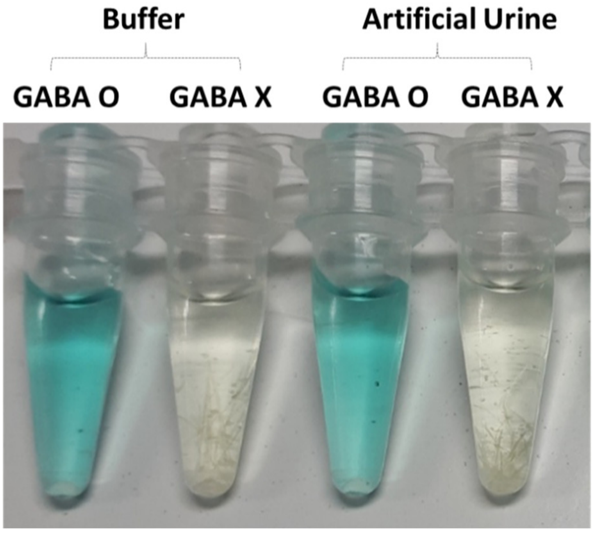

Figure S8. Image of reaction solutions containing TMB, H_2O_2, and Mn:N-CDs, that are mixed with GABA containing buffer or artificial urine, individually.

10. Detection limit of the Mn:N-CD-based sensor system for GABA detection

When the absorbance change at 652 nm of the reaction solution was monitored, the absorption variation induced by GABA was recognizable from GABA concentration of 50 nM. Then, the absorbance decreases proportionally with the increasing on GABA concentration. However, the absorbance change of the solution was not observed when the added GABA concentration is higher than 400 nM.

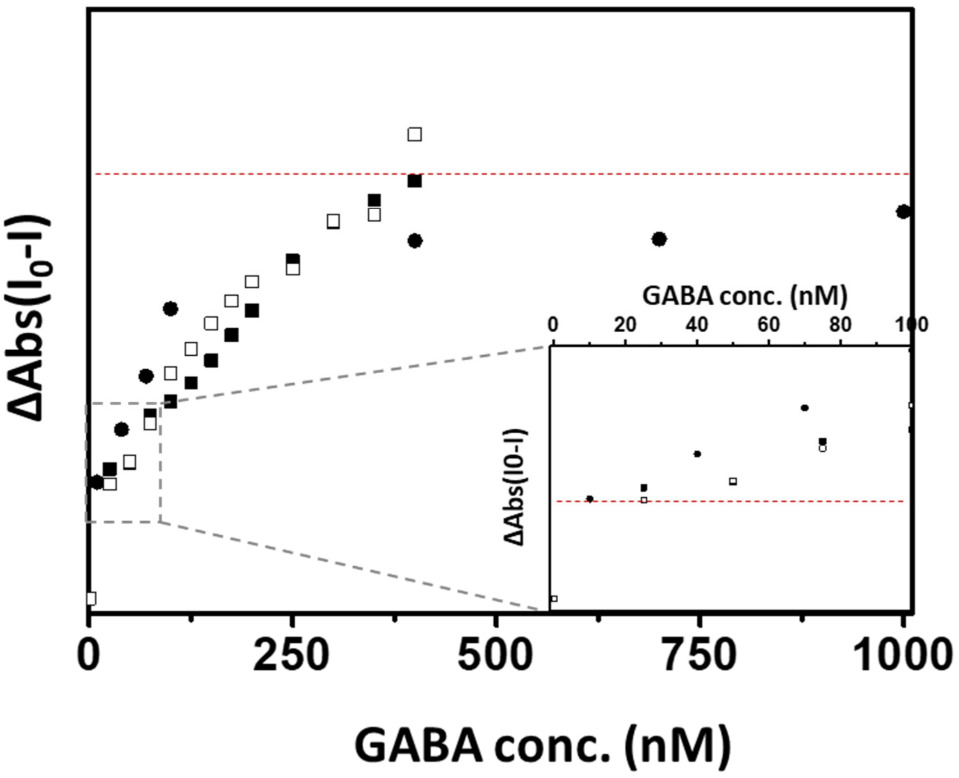

Figure S9. Variation in absorption (at 652 nm) of the mixed solution containing Mn:N-CDs, GABA, TMB, and H_2O_2 as a function of the concentration of GABA.

The limit of detection (LOD) and quantitation (LOQ) was calculated as below:

LOD = 3.3(s/S)

LOQ = 10(s/S)

s = standard deviation of the response

S = slope of the calibration curve

On average, LOD and LOQ were calculated as 59.191 nM and 179.366nM, respectively.

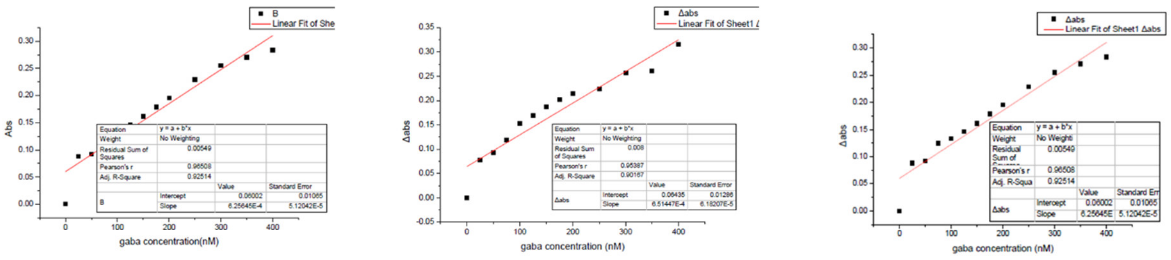

LOD=3.3(stdev/Slope)
=3.3 X (0.01065/6.256445*10^{-4})
=56.256 nM

LOQ=10(stdev/Slope)
=170.473 nM

LOD=3.3(stdev/Slope)
=3.3 X (0.01065/6.25645*10^{-4})
=65.144 nM

LOQ=10(stdev/Slope)
=197.4 nM

LOD=3.3(stdev/Slope)
=3.3 X (0.01285/6.51447*10^{-4})
=56.174 nM

LOQ=10(stdev/Slope)
=170.224 nM

Figure S10. Calculation of limit of detection (LOD) and quantitation (LOQ).
